# Gene signatures based on therapy responsiveness provide guidance for combined radiotherapy and chemotherapy for lower grade glioma

**DOI:** 10.1111/jcmm.15145

**Published:** 2020-03-11

**Authors:** Guangqi Li, Yuanjun Jiang, Xintong Lyu, Yiru Cai, Miao Zhang, Guang Li, Qiao Qiao

**Affiliations:** ^1^ Department of Radiation Oncology the First Hospital of China Medical University Shenyang China; ^2^ Department of Urology the First Hospital of China Medical University Shenyang China

**Keywords:** chemosensitivity, immunotherapy, patient stratification, radiosensitivity, risk model, TCGA, transcriptome

## Abstract

For a long time, the guidance for adjuvant chemoradiotherapy for lower grade glioma (LGG) lacks instructions on the application timing and order of radiotherapy (RT) and chemotherapy. We, therefore, aimed to develop indicators to distinguish between the different beneficiaries of RT and chemotherapy, which would provide more accurate guidance for combined chemoradiotherapy. By analysing 942 primary LGG samples from The Cancer Genome Atlas (TCGA) and the Chinese Glioma Genome Atlas (CGGA) databases, we trained and validated two gene signatures (Rscore and Cscore) that independently predicted the responsiveness to RT and chemotherapy (Rscore AUC = 0.84, Cscore AUC = 0.79) and performed better than a previous signature. When the two scores were combined, we divided patients into four groups with different prognosis after adjuvant chemoradiotherapy: RSCS (RT‐sensitive and chemotherapy‐sensitive), RSCR (RT‐sensitive and chemotherapy‐resistant), RRCS (RT‐resistant and chemotherapy‐sensitive) and RRCR (RT‐resistant and chemotherapy‐resistant). The order and dose of RT and chemotherapy can be adjusted more precisely based on this patient stratification. We further found that the RRCR group exhibited a microenvironment with significantly increased T cell inflammation. In silico analyses predicted that patients in the RRCR group would show a stronger response to checkpoint blockade immunotherapy than other patients.

## 1 INTRODUCTION

Lower grade gliomas (LGGs) in the brain arise from neuroepithelial tissue and include World Health Organization (WHO) grade II/III gliomas. These types of gliomas exhibit highly variable clinical behaviour and are therefore more difficult to predict.^1^ Their highly invasive nature precludes the possibility of complete neurosurgical resection, which indicates that postoperative adjuvant therapy is especially important. Historically, RT was the primary treatment of unresectable and progressive LGG; however, the trend shifted towards delaying RT because of the long‐term toxic side effects,^2^ especially in the treatment of chemo‐sensitive tumour types such as 1p19q‐codeleted oligodendrogliomas.^3^, ^4^, ^5^, ^6^ As the importance of chemotherapy in glioma treatment increases,^7^, ^8^ RT alone has not been an option for adjuvant treatment since the 2015 version of the NCCN Guidelines of Central Nervous System Cancers.^9^ However, pre‐RT chemotherapy with delayed RT leads to worse event‐free survival compared with immediate application of RT during the early stage of treatment.^10^, ^11^ Weighting the risk of RT against the risk of tumour progression has become the key to the patient‐specific decision‐making. In this situation, if we can predict the patient's response to different treatments separately, we can distinguish between those who would benefit from RT and those who would benefit from chemotherapy. This will not only lead to a more accurate patient selection but also guide the application timing and order of RT and chemotherapy in cases treated with combination therapy.

With the development of radiomics and genomics technology, new progress has been made in the typing and prediction of gliomas. Based on the computerized tomography (CT) and magnetic resonance imaging (MRI), various image analysis techniques have been used to predict the classification, molecular status and prognosis of LGG.^12^, ^13^, ^14^, ^15^ As a non‐invasive method, those techniques have promising application prospects. In recent years, several predictive signatures based on gene expression profiling have also been developed and performed well in predicting treatment response.^16^, ^17^, ^18^, ^19^ Different from clinical performance and molecular typing used in the NCCN Guidelines, a treatment‐related gene signature is developed to directly predict the responsiveness to a specific treatment. Due to the overlapping mechanisms of RT and chemotherapy resistance,^20^, ^21^, ^22^, ^23^ previous signatures centred on a single treatment are not dedicated to distinguishing between the different beneficiaries of RT and chemotherapy. Faced with this situation, we hope to establish new indicators to guide patient stratification and provide guidance for the use of RT and chemotherapy. For patients who are resistant to both RT and chemotherapy, we aim to study the mechanism of resistance and determine suitable treatments.

## 2 MATERIALS AND METHODS

### 2.1 Lower grade glioma data sets

The The Cancer Genome Atlas (TCGA) LGG data set (containing 516 primary LGGs) was selected as the discovery cohort. The normalized level three RNA‐seq data (FPKM) were downloaded from TCGA GDC (https://portal.gdc.cancer.gov/). The clinical information of TCGA LGGs was obtained from the University of California at Santa Cruz (UCSC) Xena (https://xena.ucsc.edu/), and the IDH status, 1p/19q codeletion, MGMT and TERT promoter status were provided by Pierre Bady.^24^ The Chinese Glioma Genome Atlas (CGGA) is the largest glioma genome database in China, which provides multiple omics data and matched clinical data of over 2000 primary and recurrent samples from Chinese cohorts. The CGGA mRNAseq_693 (containing 282 primary LGGs) and mRNAseq_325 (containing 144 primary LGGs) data sets were selected as the validation cohort for the Rscore. RSEM‐normalized gene expression and clinical data were downloaded from the CGGA (http://www.cgga.org.cn/index.jsp). Batch effects were removed using an Empirical Bayes‐based approach (ComBat) implemented in the R package “SVA”. As RNAseq_693 lacks the information for the *CPA3* gene (part of the Cscore formula), we only used RNAseq_325 when validating Cscore.

### 2.2 Immune cell composition

To determine the abundance of different immune cell types, we performed MCP counter experiment 25 to all primary LGGs. A Wilcoxon's test was used to compare the difference in cell types between the RRCR group and the others. The R package “vioplot” was used to generate the violin plot, and the R package “survival” was used for the survival analysis.

### 2.3 Weighted gene coexpression network construction

The 1047 DEGs (fold change > 2 and FDR < 0.01) between the RRCR group and the other groups was calculated by “EdgeR” and were then used to construct a weighted gene coexpression network by the “WGCNA” package 26 in the 154 RRCR samples. We determined 7 as the soft power threshold, which generated a high connectivity network with scale‐free topology. The network was constructed with the same parameters mentioned in our previous study.^27^ Five modules were detected and then related to the Rscore and Cscore through an eigengene‐based Pearson correlation analysis. Hub genes of the blue module were defined as the genes with top 25% connectivity.

### 2.4 Tumour mutational burden and tumour inflammation signature score calculation

The Cancer Genome Atlas LGG simple nucleotide variation data (VarScan) were used to calculate the tumour mutational burden (TMB), which was defined as the number of mutations per megabase.

To calculate the tumour inflammation signature (TIS) scores, we renormalized the FPKM RNAseq data using 11 housekeeping genes.^18^ The log_2_(FPKM+1) of each gene was normalized by subtracting the arithmetic mean of the log_2_(FPKM+1) of the 11 reference genes. According to Ayers et al,^18^ the TIS score was computed as the weighted sum of the housekeeping‐normalized expression of the 18 genes.

### 2.5 Statistical analysis

The differentially expressed genes (DEGs) between the non‐responders and responders were determined using the R package “edgeR” with a fold change > 2 and FDR < 0.05. A univariate Cox analysis was performed by the R package “survival”. A panel of genes was determined by LASSO analysis using the optimal λ value, which was selected through 1000 cross‐validations. The multivariate Cox analysis used the LASSO panel and tr‐DFS to generate the Rscore (Cscore), which was defined as a linear combination of the regression model coefficients (*β*) multiplied by the mRNA expression level. Kaplan‐Meier survival analysis was performed using the R package “survival”. The receiver operating characteristic (ROC) curve and area under the ROC curve (AUC) at three years were calculated using the R package “survivalROC” with a Kaplan‐Meier method. The Pearson method was used for the correlation analysis. Significant P values were calculated using the Log‐Rank method. The Gene Ontology biological process (GO_BP) and Kyoto Encyclopedia of Genes and Genomes (KEGG) enrichment analyses were performed using the Database for Annotation, Visualization and Integrated Discovery (DAVID 6.7 https://david-d.ncifcrf.gov/). All statistical analyses were performed with R software (v3.6.0).

## 3 RESULTS

### 3.1 Determination of “responders” and “non‐responders” for RT and chemotherapy

Clinical characteristics of patients in TCGA and CGGA data sets are summarized in Table 1. We used the method of Panja et al^28^ to determine the “responders” and “non‐responders” to a particular treatment. For RT, the “new tumor events” and “death” after the start of adjuvant RT were defined as “treatment‐related events” and the time from the start of adjuvant RT to treatment‐related events was defined as “treatment‐related disease‐free survival time (tr‐DFS)”. If the patient did not experience a treatment‐related event, the tr‐DFS was defined as the time from the start of RT to the last follow‐up. We included the samples used in this portion according to the following criteria:

1. The patient had undergone RT but did not receive chemotherapy,
2. The primary tumour with specific RT starts time records.

**TABLE 1:** Clinical characteristics of patients in TCGA and CGGA data sets

Characteristics	TCGA LGG (n = 516)	CGGA mRNAseq 693 (n = 282)	CGGA mRNAseq 325 (n = 144)
Age (y)	42.94 ± 13.36	39.98 ± 10.59	40.67 ± 11.16
Histologic type			
Astrocytoma	194 (37.60%)	72 (25.53%)	47 (32.64%)
Oligoastrocytoma	130 (25.19%)	159 (56.38%)	62 (43.06%)
Oligodendroglioma	191 (37.02%)	51 (18.09%)	35 (24.31%)
Unknown	1 (0.19%)	0 (0.00%)	0 (0.00%)
Histologic grade			
G2	249 (48.26%)	138 (48.94%)	94 (65.28%)
G3	265 (51.36%)	144 (51.06%)	50 (34.72%)
Unknown	2 (0.39%)	0 (0.00%)	0 (0.00%)
Adjuvant therapy			
RT and chemotherapy	196 (37.98%)	146 (51.77%)	58 (40.28%)
RT without chemotherapy	96 (18.60%)	59 (20.92%)	62 (43.06%)
Chemotherapy without RT	54 (10.47%)	25 (8.87%)	4 (2.78%)
Non‐RT and non‐chemotherapy	127 (24.61%)	35 (12.41%)	6 (4.17%)
Unknown	43 (8.33%)	17 (6.03%)	14 (9.72%)

Abbreviations: CGGA, Chinese Glioma Genome Atlas; TCGA LGG, The Cancer Genome Atlas lower grade glioma.

**FIGURE 1:**
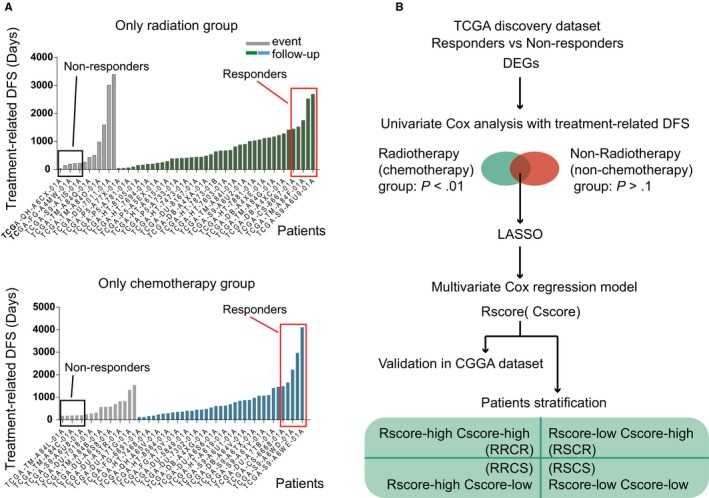
Method and process. A, Determination of responders and non‐responders to RT and chemotherapy. B, The construction process of the LASSO‐based Cox model

A total of 53 primary LGGs were selected according to the criteria and include 12 treatment‐related events and 41 follow‐ups. We ranked the 53 patients based on their tr‐DFS, and the patients who fell into the most left (10%) and right (10%) distribution tails were defined as “non‐responders” and “responders”, respectively (Figure 1A).

The “non‐responders” and “responders” to chemotherapy were determined using a similar method. Among the 51 samples that satisfied the criteria, five were defined as “non‐responders” and another five were defined as “responders” (Figure 1A).

### 3.2 Establishment and external validation of the Rscore and Cscore

**FIGURE 2:**
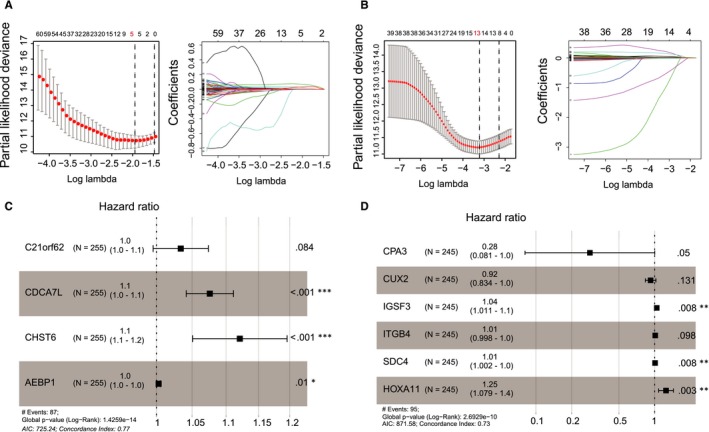
Results of LASSO and Cox analyses. A, B, Candidate genes selection by LASSO through 1000 cross‐validations of the parameter *λ*. C, D, Forest plot showing the prediction model constructed by the Stepwise Cox regression analysis using the LASSO candidate genes

We used a set of modelling processes to construct the Rscore and Cscore, which represent the degree of resistance to RT and chemotherapy, respectively (Figure 1B). A total of 255 patients in the TCGA LGG data set received radiotherapy with clear records of starting time and were included in the Rscore construction using treatment‐related disease‐free survival time (tr‐DFS). Similarly, 245 patients with clear records of starting time for chemotherapy were included in the construction of Cscore using tr‐DFS. Because the RT resistance and chemotherapy resistance share similarities in their mechanisms, we need to avoid strong collinearity between the Rscore and the Cscore to distinguish between the different beneficiaries of RT and chemotherapy. Therefore, we selected the differentially expressed genes (DEGs) between the non‐responders and responders with a *P*‐value <.01 in the RT (chemotherapy) group, and at the same time, a *P*‐value >.1 in the non‐RT (non‐chemotherapy) group in the univariate Cox analysis for tr‐DFS. These genes were called prognostic DEGs for the specific therapy and then put into a least absolute shrinkage and selection operator (LASSO)‐based multivariate Cox analysis. The LASSO selected five candidate genes from the 141 prognostic DEGs for radiotherapy through 1000 cross‐validations of the parameter *λ* (Figure 2A). Thirteen candidate genes for chemotherapy were selected from 39 prognostic DEGs (Figure 2B). The candidate genes were then put into a stepwise multivariate Cox analysis to build an optimal prediction model using tr‐DFS (Figure 2C,D). According to the regression model, we obtained the Rscore containing the expression of 4 genes and the Cscore containing the expression of 6 genes with the following formulas:

Rcore = 0.03330365 × expression level of *C21orf62* + 0.07308094 × expression level of *CDCA7L* + 0.11414216 × expression level of *CHST6* + 0.00303856 × expression level of *AEBP1*
Cscore = −1.25569210 × expression level of *CPA3* − 0.07888934 × expression level of *CUX2* + 0.04030749 × expression level of *IGSF3* + 0.01055746 × expression level of *ITGB4* + 0.00775530 × expression level of *SDC4* + 0.21961659 × expression level of *HOXA11*

According to the meaning of the risk model, the higher the two scores, the stronger the resistance to the relevant treatment. We then tested the collinearity between the Rscore and the Cscore in all primary tumours, and the Pearson correlation coefficient was only 0.25 (*P* < .0001).

**FIGURE 3:**
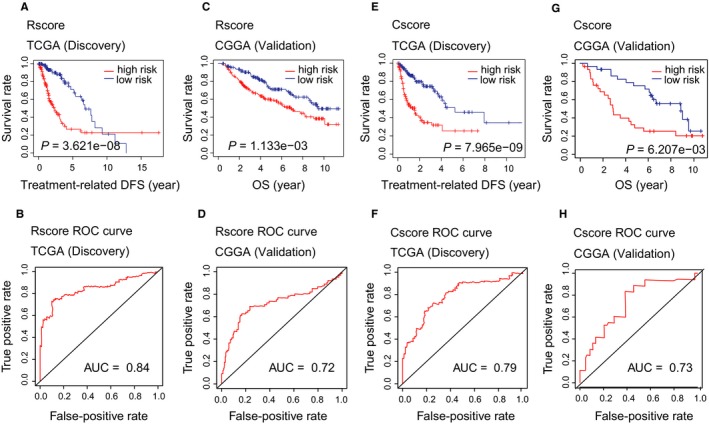
Validation of the Rscore and Cscore

We next tested the two scores in the discovery and validation sets. A total of 328 patients in the CGGA data sets received radiotherapy were used to validate Rscore and 60 patients with chemotherapy were used to validate Cscore. We divided patients into low‐ and high‐risk group based on the median Rscore (Cscore), and the patients who received the relevant therapy (RT or chemotherapy) were selected for the survival analysis with tr‐DFS. Significant survival differences were shown for high and low Rscores in the TCGA (*P* = 3.621e−08, AUC = 0.84) and CGGA (*P* = 1.133e−03, AUC = 0.72) data sets (Figure 3A‐D). The Cscore also showed similar predictive power in both the TCGA (*P* = 7.965e−09, AUC = 0.79) and the CGGA (*P* = 6.207e−03, AUC = 0.73) data sets (Figure 3E‐H).

To remove the effect of other confounding factors such as histologic grade, patient age, whether RT or chemotherapy was given, Karnofsky performance score, IDH status, 1p/19q codeletion, MGMT and TERT promoter status,^1^, ^29^, ^30^, ^31^, ^32^ we performed a multivariate Cox regression analysis using tr‐DFS to test the independence of our models after controlling all these confounding factors. The results showed that both the Rscore and the Cscore were independent prognostic factors (Rscore *P* = .03, Cscore *P* = .01, Figure S1A,B).

Prior to this study, a radiosensitivity predictive assay (RSI)^16^ was widely used to predict tumour response to RT; this score was then compared with the Rscore. The results showed that RSI could not predict the RT‐related DFS or overall survival (OS) of patients in the TCGA LGG data set who received RT (Figure S1C,D). However, RSI could predict the OS of patients in CGGA LGG data set who received RT (*P* = 2.589e−03), and the AUC was less than the Rscore (0.62 vs 0.72) (Figure S1E,F).

### 3.3 Patient stratification based on the Rcore and Cscore

According to the Rcore and Cscore levels, we divided the TCGA primary LGGs into four groups (6 patients without FPKM data were ignored): RSCS (RT‐sensitive and chemotherapy‐sensitive, 169 samples), RSCR (RT‐sensitive and chemotherapy‐resistant, 99 samples), RRCS (RT‐resistant and chemotherapy‐sensitive, 88 samples) and RRCR (RT‐resistant and chemotherapy‐resistant, 154 samples) (Figure 1B). The median scores were used as the cut‐off values. The clinical information of the four groups is shown in Table 2.

**TABLE 2:** Clinical characteristics of patients in the four groups defined by Rscore and Cscore

Characteristics	RSCS (n = 169)	RSCR (n = 99)	RRCS (n = 88)	RRCR (n = 154)
Age (y)	41.06 ± 12.80	40.16 ± 12.64	44.75 ± 13.00	45.56 ± 13.98
Histologic type				
Astrocytoma	52 (30.77%)	40 (40.40%)	25 (28.41%)	75 (48.70%)
Oligoastrocytoma	36 (21.30%)	28 (28.28%)	20 (22.73%)	44 (28.57%)
Oligodendroglioma	80 (47.34%)	31 (31.31%)	43 (48.86%)	35 (22.73%)
Unknown	1 (0.59%)	0 (0.00%)	0 (0.00%)	0 (0.00%)
Histologic grade				
G2	86 (50.89%)	58 (58.59%)	39 (44.32%)	64 (41.56%)
G3	82 (48.52%)	40 (40.40%)	49 (55.68%)	90 (58.44%)
Unknown	1 (0.59%)	1 (1.01%)	0 (0.00%)	0 (0.00%)
IDH status				
Mutant	161 (95.27%)	89 (89.90%)	78 (88.64%)	85 (55.19%)
WT	8 (4.73%)	9 (9.09%)	10 (11.36%)	67 (43.51%)
Unknown	0 (0.00%)	1 (1.01%)	0 (0.00%)	2 (1.30%)
1p/19q codeletion				
Non‐codeletion	96 (56.80%)	80 (80.81%)	38 (43.18%)	129 (83.77%)
Codeletion	73 (43.20%)	19 (19.19%)	50 (56.82%)	25 (16.23%)
Unknown	0 (0.00%)	0 (0.00%)	0 (0.00%)	0 (0.00%)
MGMT promoter				
Unmethylated	17 (10.06%)	17 (17.17%)	8 (9.09%)	48 (31.17%)
Methylated	152 (89.94%)	82 (82.83%)	80 (90.91%)	106 (68.83%)
Unknown	0 (0.00%)	0 (0.00%)	0 (0.00%)	0 (0.00%)
TERT promoter				
Mutant	39 (23.08%)	13 (13.13%)	31 (35.23%)	47 (30.52%)
WT	54 (31.95%)	47 (47.47%)	23 (26.14%)	34 (22.08%)
Unknown	76 (44.97%)	39 (39.39%)	34 (38.64%)	73 (47.40%)

Abbreviations: RRCR, RT‐resistant and chemotherapy‐resistant; RRCS, RT‐resistant and chemotherapy‐sensitive; RSCR, RT‐sensitive and chemotherapy‐resistant; RSCS, RT‐sensitive and chemotherapy‐sensitive.

**FIGURE 4:**
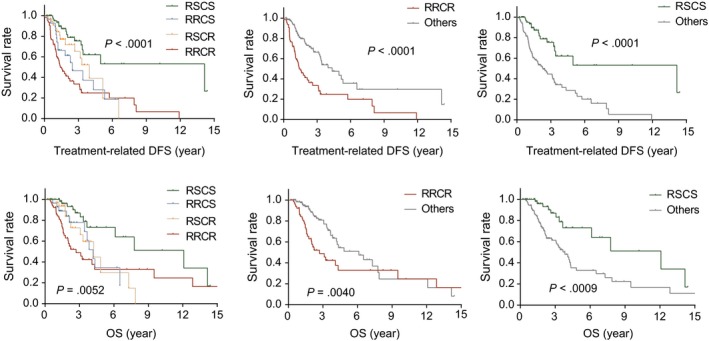
Stratification based on the Rscore and Cscore was validated to predict treatment response

Although the predictive power of the two scores in relevant patients has been independently verified in the previous section, we needed to further validate the predictive ability of the stratification based on the combination of the two scores. We selected the 194 patients in the TCGA data set who received both RT and chemotherapy for the OS analysis. The results showed that the 4 groups had significantly different survival rates and that the RRCR and RSCS groups exhibited significantly lower and higher survival rates, respectively, than the other groups (Figure 4). Similar results were also observed in the CGGA cohort (Figure S1G‐I).

### 3.4 A higher proportion of CD8^+^ T cells and B cells correlated with therapy resistance in the RRCR group

**FIGURE 5:**
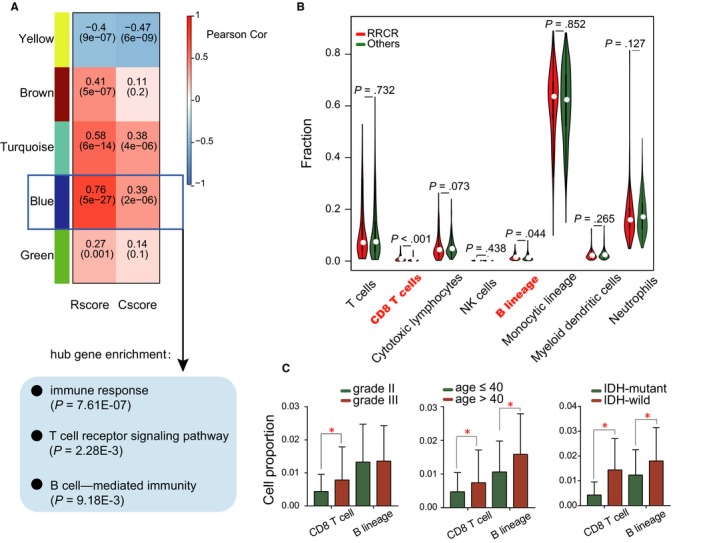
The immune microenvironment of the RRCR group. A, WGCNA determined a coexpression module highly correlated to the Rscore and Cscore. Enrichment analysis showed that the module was related to immune response. B, Comparison of immune cell proportions between the RRCR and other groups. C, CD8 T cell and B lineage cell proportions were related to histologic grade, patient age and IDH status

To further investigate the mechanism of therapy resistance in the RRCR group, we performed a weighted gene coexpression network analysis in the 154 RRCR patients using the 1047 DEGs (fold change > 2, FDR < 0.01) between the RRCR and the other groups. A blue module containing 180 genes was detected to show the highest correlation to Rscore (Pearson cor = .76, *P* = 5E‐27) and Cscore (Pearson cor = .39, *P* = 2E−06) (Figure 5A). A gene enrichment analysis showed that the hub genes (top 25% connectivity) in this module were associated with immune response, T cell receptor signalling and B cell lineage–mediated immunity.

The results of the WGCNA indicated that the immune microenvironment of the RRCR group might differ from that of the other groups, and the immune infiltration positively correlated to therapy resistance. To examine the immune cell differences between the RRCR and other groups, we applied an MCP counter to all primary LGG samples. Compared with other groups, the RRCR group showed a significantly higher proportion of CD8^+^ T cells and B lineage cells (Figure 5B), which was consistent with the results obtained by the WGCNA.

We further found that the proportions of CD8 T cells and B lineage cells were correlated with histologic grade, age and IDH status. In IDH‐wild‐type gliomas and patients >40 years of age, the proportions of CD8 T cells and B lineage cells were significantly increased. A higher CD8 T cell proportion was also correlated with higher histologic grade (Figure 5C), which was demonstrated in a previous study.^33^

It should be noted that the TCGA and CGGA gene expression profiles were derived from tissue sequencing experiments prior to treatment. Therefore, the cell type proportions that were simulated based on gene expression profiles were pre‐treatment profiles and were not affected by subsequent treatment.

### 3.5 The RRCR group was more suited for checkpoint blockade immunotherapy than other groups

The T cell–inflamed tumour microenvironment has become a biomarker for checkpoint blockade immunotherapy response.^34^ As the RRCR group had a significantly higher infiltration of CD8 T cells, we wondered whether this group of patients would be more suited for checkpoint blockade immunotherapy than other groups.

Two categories of biomarkers predict the response to checkpoint blockade immunotherapies: biomarkers related to tumour neoepitope burden and biomarkers indicative of a T cell–inflamed tumour microenvironment.^34^ The former includes microsatellite instability (MSI) and tumour mutational burden (TMB), while the latter includes the tumour inflammation signature (TIS)^18^ and the expression of multiple inhibitory receptors (IRs) such as programmed cell death ligand‐1 (PD‐L1) and cytotoxic T lymphocyte associated protein 4 (CTLA4).

**FIGURE 6:**
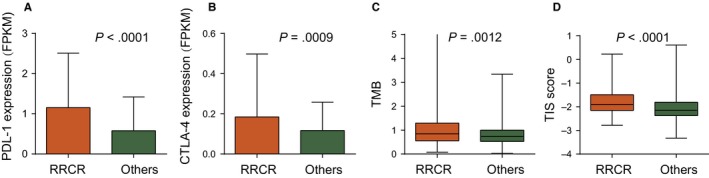
Comparison of PD‐L1, CTLA4 expression as well as TMB and TIS between the RRCR and the other groups

The expression of PD‐L1 and CTLA4 was up‐regulated in the RRCR group compared with the other groups (Figure 6A,B), which suggests that both anti‐PD1 and anti‐CTLA4 treatments in the RRCR group might lead to response. We then calculated the TMB and TIS of the RRCR group and the other groups. As shown in Figure 6C,D, both TMB (*P* = .001) and TIS (*P* < .0001) were significantly higher in the RRCR group than in the other groups. These results indicated that the patients in the RRCR group would exhibit a stronger response to checkpoint blockade immunotherapy than patient in the other groups.

## 4 DISCUSSION

To distinguish between those who would benefit from RT and chemotherapy and to provide more accurate guidance for patient selection and treatment timing, we developed and tested the Rscore and Cscore using 942 LGGs. Considering the convenience of clinical application, we chose the Cox proportional hazard model, a semi‐parametric model which generates a comparable risk score with a concise formula, to predict the risk. Due to the high dimensionality of transcriptome data, we added LASSO for feature selection. The LASSO is a popular method for regression of high‐dimensional features, which has been widely applied to the Cox model for survival analysis of high‐dimensional data.^35^, ^36^, ^37^ During the process of model construction, we used treatment‐related DEGs and treatment‐specific risk genes to control the therapeutic specificity of the model. The two scores have been demonstrated to be independent predictors of treatment response. Prior to this, a radiotherapy sensitivity indicator called RSI has been validated in a variety of cancers 16, 38, 39, 40, 41 and has recently been further developed.^42^, ^43^ However, the predictive power of RSI is lower than Rscore in TCGA or CGGA LGG data sets. The result of this comparison suggests that RSI, as a cross‐tumour indicator, is still tumour type‐dependent and does not ideally predict RT response in patient with LGG. Tumour‐specific and treatment‐related signatures such as the Rscore and Cscore may perform better in this situation. As the formula shows, to calculate Rscore and Cscore we only need to detect the expression of 4 and 6 genes, respectively, which is convenient for clinical application.

The low collinearity of the two indicators allowed us to combine them for patient stratification. Patients in the RSCS group are sensitive to both RT and chemotherapy and may benefit from combined chemoradiotherapy with high survival rates. However, patients in the RRCS group and RSCR group are sensitive to one of the two adjuvant treatments and are resistant to the other one. We believe that the response differences to RT and chemotherapy would provide guidance for more precise adjuvant chemoradiotherapy, including adjustments to the application timing, order, intensity and period of RT and chemotherapy. Further clinical trials are needed to verify whether the treatment design based on this stratification can improve the efficacy of the adjuvant chemoradiotherapy and reduce the side effects.

We found that the resistance in the RRCR group was significantly correlated with the infiltration of CD8 T cells and B lineage cells. Patients in the RRCR group presented a microenvironment highly infiltrated by T cells. An in silico analysis based on tumour mutational burden (TMB), tumour inflammation signature (TIS) and expression of inhibitory receptors (IRs) predicted that patients in the RRCR group would show a stronger response to checkpoint blockade immunotherapy than patients in the other groups. This result suggests that in addition to traditional postoperative RT and chemotherapy, checkpoint blockade immunotherapy may be added to the regimen of the RRCR group, which requires further verification by clinical studies. For each single patient, anti‐PD1 or anti‐CTLA4 therapy can be chosen according to the expression of PDL‐1 and CTLA4.

It should be noted that this study still has some limitations. Although the resistance mechanisms of different chemotherapeutic drugs are highly overlapping, as a universal drug resistance index, the Cscore may not perform as well when predicting the response to a specific drug. In addition, 78% of the patients in the TCGA data set who received chemotherapy were treated with temozolomide (TMZ), while PCV and other drugs were much less frequently used in this data set, which suggests that the Cscore is less representative of other drugs.

## 5 CONCLUSIONS

Patient stratification based on the Rscore and Cscore can be used to guide the clinical application of RT and chemotherapy in patients with LGG. For the patients who show resistance to both RT and chemotherapy, additional checkpoint blockade immunotherapy is recommended.

## CONFLICT OF INTEREST

The authors declare that they have no conflicts of interest.

## AUTHOR CONTRIBUTIONS

GQ.L. and QQ have made substantial contributions to conception and design; GQ.L., YJ.J., XT.L., YR.C and MZ have made substantial contributions to acquisition of data, or analysis and interpretation of data. GQ.L., GL and QQ have been involved in drafting the manuscript or revising it critically for important intellectual content. All authors have given final approval of the version to be published.

## Supporting information


**Figure S1**

## Data Availability

All data sets used in this study are described in detail in Methods and Materials.
