# Supplementary figures and images for: Gene signatures based on therapy responsiveness provide guidance for combined radiotherapy and chemotherapy for lower grade glioma
Source: J Cell Mol Med. 2020 Mar 11;24(8):4726–35. doi: 10.1111/jcmm.15145 (PMC7176846; doi:10.1111/jcmm.15145)

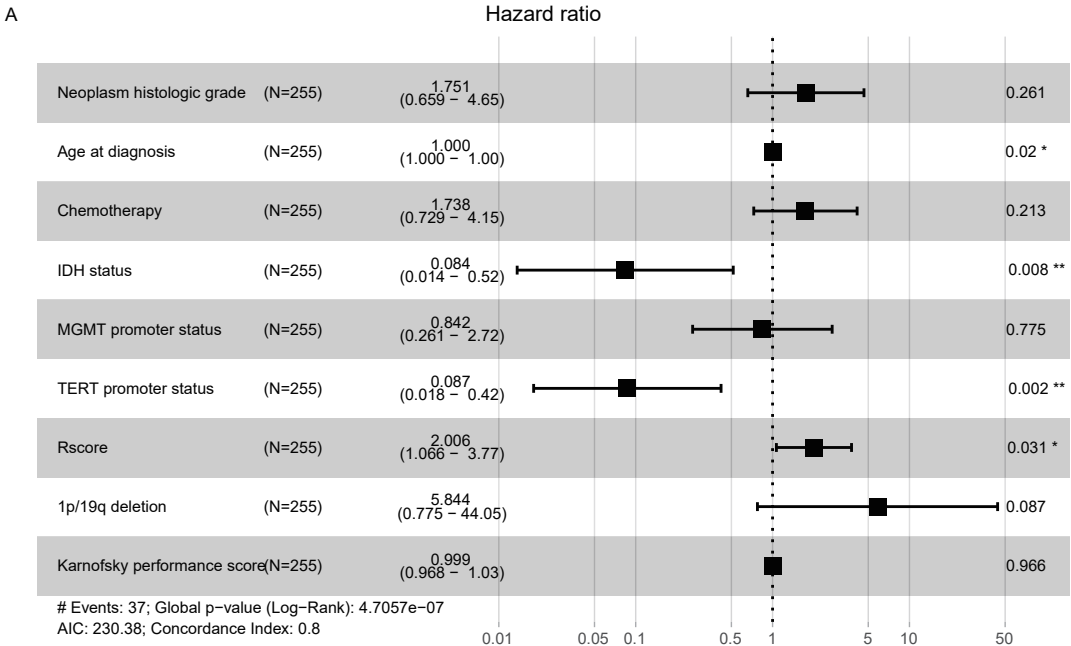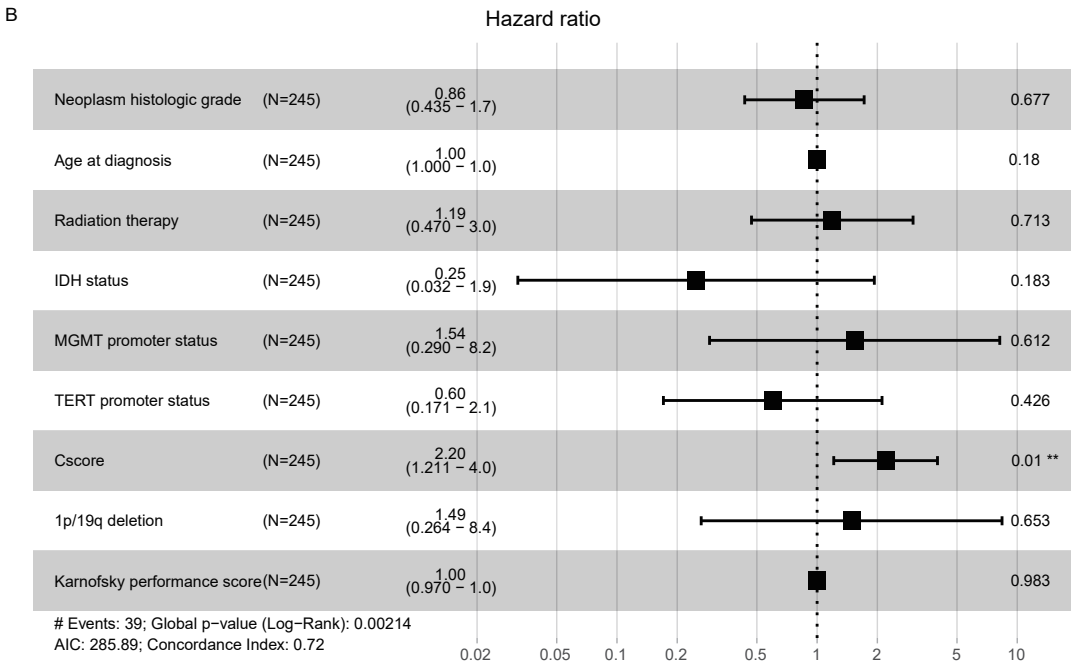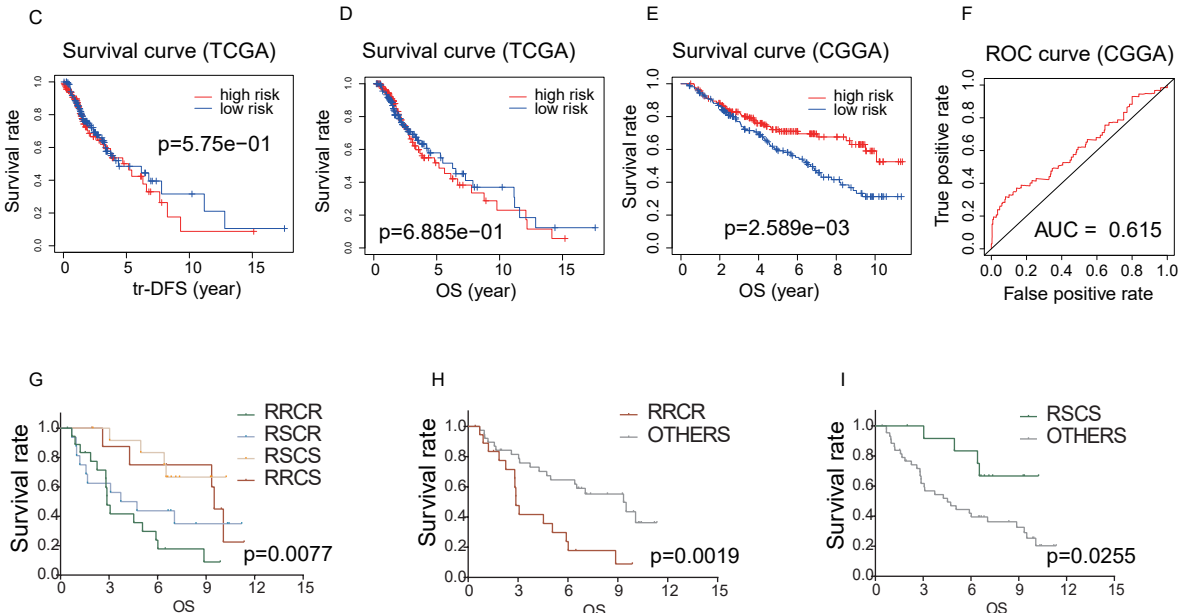

Supplement: Supplementary file 1 — Figure S1 [file JCMM-24-4726-s001.pdf]
